# Supplementary material for: The Ultratrace Determination of Fluoroquinolones in River Water Samples by an Online Solid-Phase Extraction Method Using a Molecularly Imprinted Polymer as a Selective Sorbent
Source: Molecules. 2022 Nov 22;27(23):8120. doi: 10.3390/molecules27238120 (PMC9737498; doi:10.3390/molecules27238120)
Supplement: Supplementary file 1 [file molecules-27-08120-s001.zip › molecules-2026946-Supplementary.pdf]

Table S1. Chromatographic mobile phase gradient.

Time (min)	TFA (0.5%; pH 2.0)	ACN (%)	MeOH (%)
0.0	90	10	0
10.0	90	10	0
20.0	80	20	0
30.0	74	6	20
35.0	52	8	40
40.0	70	30	0
50.0	55	45	0
50.1	10	90	0
60.0	10	90	0
